# CD18 Mediates Neutrophil Imperviousness to the *Aggregatibacter actinomycetemcomitans* JP2 Clone in Molar-Incisor Pattern Periodontitis

**DOI:** 10.3389/fimmu.2022.847372

**Published:** 2022-05-18

**Authors:** Koren Hashai, Ian L. Chapple, Lior Shapira, Walaa Assadi, Stav Dadon, David Polak

**Affiliations:** ^1^ Faculty of Dental Medicine, Hebrew University of Jerusalem, Jerusalem, Israel; ^2^ Department of Periodontics, Hadassah Medical Center, Jerusalem, Israel; ^3^ Institute of Clinical Sciences, College of Medical and Dental Sciences, School of Dentistry, University of Birmingham, Birmingham, United Kingdom; ^4^ Birmingham Community Healthcare National Health Service (NHS) Foundation Trust, Birmingham, United Kingdom; ^5^ Department of Orthodontics, Rambam Medical Center, Haifa, Israel; ^6^ Department of Orthodontics, Hadassah Medical Center, Jerusalem, Israel

**Keywords:** neutrophils (PMNs), periodontitis (inflammatory), CD18, oxidative stress, JP2 clone

## Abstract

**Introduction:**

Molar-incisor pattern periodontitis (MIPP) in the absence of significant local risk factors or systemic disease, is a rare, early onset periodontal disease phenotype, with 0.5% to 2.5% global prevalence. The condition is characterized by impaired neutrophil function and persistent *Aggregatibacter actinomycetemcomitans* (JP2 clone) infection. The aim of this study was to characterize neutrophil functional responses to JP2 and to investigate the neutrophil receptors involved.

**Materials and Methods:**

Neutrophils were obtained from whole blood samples of periodontally healthy and MIPP subjects and incubated with the JP2 clone or a non-JP2 clone of *A. actinomycetemcomitans*. Bacterial survival was tested by blood agar culture; neutrophil death was tested with propidium iodide and flow cytometry; Reactive oxygen production (ROS) was measured with 2′,7′-dichlorofluorescein diacetate and a fluorescence plate reader; the cytokinome was analysed using an array profiler, ELISA and RT-PCR. Receptors binding to JP2 were isolated using a novel immunoprecipitation assay and validated functionally using specific blocking antibodies.

**Results:**

JP2 and non-JP2 survival was comparable between all the neutrophil groups. Resistance to neutrophil necrosis following exposure to JP2 was significantly lower in the MIPP group, than in all the other groups (p<0.0001). Conversely, MIPP neutrophils showed lower levels of ROS production in response to JP2 infection compared with that of healthy neutrophils (p<0.001). Furthermore, significantly lower levels of cytokines, such as IL8, IL10 and TNFα, were observed during JP2 incubation with MIPP neutrophils than upon incubation with periodontally healthy neutrophils. Various proteins expressed on neutrophils bind to JP2. Of these, CD18 was found to mediate neutrophil necrosis. The CD18 receptor on MIPP neutrophils acts differently from that on periodontally healthy patients neutrophils, and appears to reflect differential neutrophil reactions to JP2.

**Conclusion:**

This study portrays a fundamental difference in neutrophil response to JP2 infection between periodontally healthy and MIPP patients. This was evident in the resistance to necrosis, and lower ROS and cytokine production, despite the persistent presence of viable JP2. Whilst in periodontally healthy neutrophils, JP2 binds to CD18 on cell surfaces, this is not the case in MIPP neutrophils, suggesting a potential role for CD18 in the periodontal susceptibility of MIPP patients.

## Introduction

Molar-incisor pattern periodontitis (Periodontitis, grade c, where bone loss is inconsistent with levels of plaque (formally known as localized aggressive periodontitis)) is a rare form of the disease affecting less than 0.5% of the overall population (1, 2). However, in some parts of the world, its prevalence may rise to over 6% (2, 3). Aside from the age at onset, the location of the lesions, and the rapidity of periodontal tissue breakdown, it typically appears in individuals that are otherwise systemically healthy and destruction is inconsistent with levels of plaque control (4, 5). The host response plays a key pathogenic role in MIPP (5–7) and there is evidence of a link between MIPP susceptibility and altered neutrophil function. However, MIPP patients are otherwise healthy and are not susceptible to systemic infections, implying that dysregulated immune-inflammatory responses are locally induced/active.

The subgingival microbiota of MIPP subjects indicates a unique collection of organisms that differs significantly from that of periodontally heathy individuals. Of those microorganisms, the highly virulent JP2 genotype of *A. actinomycetemcomitans* (JP2 clone) shows a strong association with MIPP pathogenesis (8). Furthermore, 78%- 83% of African American children diagnosed with MIPP posess the JP2 genotype (8, 9).

*A. actinomycetemcomitans* possesses various virulence factors that modulate host defense activity. Among these, the production of leukotoxin A (Ltx), which is expressed on the bacterial cell wall and also secreted, has been highlighted as a key virulence factor. This cell-specific leukotoxin induces cytolysis of human polymorphonuclear leukocytes and peripheral monocytes, thereby disabling host immune defenses and allowing the microbe to evade destruction (8, 10). Whereas all *A. actinomycetemcomitans* clones maintain a similar transcriptional organization, the JP2 Ltx operon possesses a unique 530 base pair deletion sequence that sanctions robust Ltx production (10 to 20 times more than the non-JP2 strain) (8, 11).

Lymphocyte function-associated antigen-1 integrin (LFA-1) is believed to be the functional receptor for Ltx. LFA-1 is a transmembrane glycoprotein on the leukocyte surface that serves both as an adhesion molecule and signaling receptor. It is one of the four members of the β2-integrin family and is made up of two non-covalently linked subunits: CD11a and CD18 (12). In particular, the CD18 intermediate area is required for leukotoxin binding and the toxic effect on lymphocytes (13).

Neutrophils (neutrophilic polymorphonuclear leukocytes; PMNs) employ several mechanisms to kill invading pathogens, including phagocytosis, release of neutrophil extracellular traps and production of reactive oxygen species (ROS) (14, 15). They are the most abundant leukocytes in the blood and serve as the key player of the non-specific immune response, being the first line of defense against invading microorganisms, with multiple roles in maintaining body homeostasis (7, 16). They have been described as “double-edged swords” in the periodontium, capable of producing tissue damage in periodontal disease, as well as protecting against disease-associated tissue destruction (17). Studies report that compromised neutrophil function predisposes an individual to periodontitis (10, 16, 18). These findings were further corroborated by investigations into various systemic conditions that exhibited impaired peripheral neutrophil function, which also present with severe and early onset forms of periodontitis. These include Chediak–Higashi syndrome, cyclic neutropenia, leukocyte adhesion deficiency, Papillon–Lefevre syndrome (19), Down’s syndrome and diabetes mellitus (16). Such observations highlight the importance of altered neutrophil function in the pathogenesis of periodontitis (7).

Although it is known that neutrophils and JP2 are involved in the pathogenesis of MIPP, there is scant evidence regarding the interaction of MIPP neutrophils and the whole JP2-clone of A. *actinomycetemcomitans*. This study aims to provide new insights into the neutrophil-JP2 interaction in an attempt to further elaborate biological links between neutrophils and MIPP.

## Methods

The study was approved by the institutional Helsinki review board (Hadassah Medical Center approval number 0514-13-HMO). All subjects signed their inform consent before participation in the study.

### Patient Recruitment

Periodontally healthy, generalized periodontitis [stage III, grade B, (20)] and MIPP patients (cases with inderdental loss of attachment ≥5 mm at first molars and incisors and with radiographic evidence of vertical bone loss at the same sites) were recruited prior to periodontal treatment from the Periodontal Department, Hebrew University- Hadassah, Ein Karem between 2017-2019. Periodontal diagnosis was determined using radiographs and detailed periodontal clinical charts. Inclusion criteria included: patients 18y old or older, generally healthy and willing to participate in the study. Exclusion criteria included: smoking, pregnancy and lactating, antibiotic consumption within 6 months of participation, drug/alcohol abuse, and chronic consumption of any drug.

### Primary Neutrophils

Cells were obtained on the morning of the experiments from whole blood in standard Lithium Heparin vacutainer tubes. Neutrophils were isolated from whole blood by density gradient centrifugation (Polymorphprep, Axis-shield, Oslo, Norway), according to the manufacturer’s instructions. In brief, blood was layered on an equal volume of Polymorphprep in a 15ml tube and centrifuged for 30min, 500g at room temperature (RT). The lower cellular white band contained PMNs and was separated, eluted with red blood cells lysis buffer (Invitrogen) and centrifuged for 10 min, 400g at RT.

### Bacteria

#### *A. actinomycetemcomitans* 275253 and the Highly Leukotoxic *A. actinomycetemcomitans* JP2 Clone

A clinical isolate of JP2 was kindly provided by Prof. Gilad Bachrach, Hebrew University. Its purity was validated with TVSB selective culture (21) and PCR of the bacterium’s leukotoxin promoter region (22). Both *A. actinomycetemcomitans* and JP2 were incubated in broth containing: 0.5g yeast extract, 1.5g tryptone, 0.74g D-glucose, 0.25g NaCl, 0.075g L-cysteine, 0.05g sodium thioglycolate, 4% NaHCO_3_ (all purchased from Sigma–Aldrich, Rehovot, Israel) per litre double distilled water. The bacteria were grown in a 37°C, 5% CO_2_ chamber. Bacterial concentration was standardized according to Mattiello, Coelho, Martins, Mattiello, & Ferrao Junior (23) as OD640nm =0.1 *A. actinomycetemcomitans*/JP2 corresponding to 1.5 x 10^8^bacteria/ml.

#### *P. gingivalis* ATCC 33277 (Pg)

Pg cells were Incubated in “Wilkins” broth (Chagren broth, Oxid Ltd., UK), 37^0^C under anaerobic conditions (10% CO_2_, 5%H_2_, 85% N_2_(. Bacterial purity was validated with phase microscopy. Bacterial concentration was standardized on the basis of optical density according to Mattiello, Coelho, Martins, Mattiello, & Ferrao Junior (23) as OD650nm = 0.1, corresponding to 10^10^ bacteria/mL.

### Neutrophil Cytotoxicity Assay

Neutrophils were incubated with the different bacteria (A. *actinomycetemcomitans*, JP2 or Pg) in a ten-fold multiplicity of infection (MOI 10) in a 24 well plate (5 × 10^5^ cells/well) for 90min at 37°C. After incubation samples were transferred to collection tubes and centrifuged (7min/25^0^c/1500 rpm). The supernatants were then decanted and the cells were resuspended in 100 µL PBS and 5 µL propidium iodide (PI, Sigma) was added to each sample. After 15 min incubation in the dark, the samples were analyzed by flow cytometry (BD Accuri™ C6 Cytometer, San Jose, CA, USA). The experiments were performed in triplicate for 8 different patients with MIPP and 8 healthy controls.

### Reactive Oxygen Species (ROS) Assay

Neutrophils (5 × 10^4^ cells/well) from healthy controls and MIPP patients were incubated with A. *actinomycetemcomitans* or JP2 at a MOI-10 ratio in a 96-well black plate (PerkinElmer, Waltham, MA). HOCl (0.0006%) served as positive control. DCFH-DA (7′-dichlorofluorescein diacetate, Sigma–Aldrich, Israel) was added to each well as a marker for ROS. Immediately after addition of the bacteria, the plate was transferred to a florescence plate reader (infinite plate reader; Tecan, Männedorf, Switzerland) at 37˚C with readings (490nm excitation and 535nm emission) every 5 min for a total 150min.

The experiments were repeated in triplicate for 8 MIPP patients and 8 healthy controls.

### Bacterial Survival Assay

Neutrophils were incubated with the bacteria (5 × 10^5^ cells/well; MOI-10) in 24 well plates. After 90 min, samples from all the groups were seeded on 5% sheep blood agar (Hay Labs, Rehovot, Israel) in eight 10-fold dilutions. The plates were incubated at 37°C for one week in a 5% CO_2_ chamber, after which colonies were counted and colony forming units (CFU) were calculated.

The experiments were repeated in triplicate for 8 patients with MIPP and 8 healthy controls.

### Sample Preparation for Cytokine Analysis

Similarly to the above assays, neutrophils were incubated with the different bacteria for 3 hrs (verses un-exposed naïve controls) and centrifuged at 1200RPM at RT for 20min. The supernatants were collected and stored for protein-based analysis and the pellets were eluted in Trisol (Invitrogen) for RT-PCR analysis. All samples were stored at -80^0^ C until analysis.

### Profiler Array

A human cytokine array kit, hematopoietic receptor and non-hematopoietic receptor array kits (all from R&D systems, Minneapolis, MN, USA), were used according to the manufacturer’s instructions.

For the cytokinome assay, pooled samples from all the cases (equal volumes) were mixed by groups (MIPP and periodontally healthy), separately and used according to the manufacturer’s instruction.

For the neutrophil receptor assay, HL60 cells (Human acute promyelocytic leukemia cell line 60) were lysed in EDTA lysis buffer (10mM Tris-HCl, 2mM EDTA, 1% SDS in PBS) supplemented with protease inhibitors (Sigma), centrifuged at 25,000g at 4°C for 10 min and incubated with JP2 (at MOI 10) for 20min at RT. Then the lysates with the JP2 and adhering proteins were centrifuged at 4000 RPM for 20 min at RT and resuspended in stripping buffer ​(15 g/l glycine, 1 g/l SDS, 1% Tween 20 in DDW) for 10 min at RT to detach the proteins. After 0.22µm filtration to remove the JP2, the eluted proteins were used for the arrays.

“Image lab” (BIO-RAD, Hercules, CA, USA) software was used for semi-quantification of each protein by dot size and intensity.

### ELISA

Four cytokines, IL8, IL10, TNFα, mCSF (all from R&D Systems), chosen according to the results of the cytokine profiler, were further validated using ELISA. Samples were analyzed as individual values for each patient. In brief, ELISA plates were coated with capture antibody overnight at RT. The plates were then washed with wash buffer (0.05% tween diluted 20 x in PBS) and blocked with 1% bovine serum albumin-PBS) for 1 hr at RT. The plates were then washed, and samples and standards were added for a further 2 hrs incubation at RT. Secondary antibodies, followed by streptavidin-HRP and substrate solution (TMB, R&D systems) were then used and the reaction was terminated by the addition of 2N H_2_SO_4_. The results were analyzed with a spectrophotometer (ELISA reader) at 540nm wavelength.

### Real-Time PCR Analysis

Similarly to the ELISA assay, samples were analyzed for each patient. RNA was extracted from the cell samples with the TRIzol™ Reagent (Invitrogen), according to the manufacturer’s instructions. The purified RNAs were translated into cDNA with a QuantaBio kit (Beverly, MA, USA(according to the manufacturer’s instructions and the synthesized cDNAs were amplified with SYBR Green real-time PCR and specific primers for the genes of interest (ABI Prism 7300 System, Applied Biosystems, Foster City, CA, USA). The 20 μl reaction volume contained 10 μl PerfeCTa SYBR Green FastMix (Quantabio, Beverly, MA, USA), 500 nmol/L each of the forward and reverse primesr, and 5 μl cDNA. The thermal profile for SYBR Green real-time RT-PCR was 95°C for 10 min, followed by 40 cycles of 95°C for 15 sec and 58°C for 1 min.

The sequences of each of the primers and their sources are: IL8- F: ATGACTTCCAAGCTGGCCGTGGCT, R: TCTCAGCCCTCTTCAAAAACTTCTC (24); IL10: F: GCCTAACATGCTTCGAGATC, R: TGATGTCTGGG CTTGGTT (25); mCSF: F: 5′-TAGCCACATGATTGGGAGTG-3′, R: 5′-TATCTCTGAAGCGCATGGTG-3′ (26); TNFa: F: GAAAGCATGATCCGGGACGTG, R: GATGGCAGAGAGGAGGTTGAC (27); GAPDH (as house-keeping gene(: F: AGTTGGGATAGGGCCTCTCTT, R: TCCCACTCTTCCACCTTCGA (28). The results were analyzed according to the ΔΔCt protocol.

### JP2-Neutrophil Interaction

HL60 cells (ATCC) were cultured in Iscove’s Modified Dulbecco’s Medium (IMDM) supplemented with 20% fetal bovine serum. For differentiation into neutrophil-like cells, the cell medium was supplemented with 1.4% DMSO for 4 days, and they are thereafter referred to as neutrophils (29).

A 0.2% trypan blue solution was added to HL60 cells incubated with JP2, and after 15 min on ice, the cells were washed with PBS and analyzed by flow cytometry. When stated, 4% paraformaldehyde in PBS was used to fix JP2 and/or HL60 on ice for 20 min before JP2 incubation with the neutrophils. When stated, a stripping buffer was added to the HL60 cells incubated with JP2, which were then analyzed by flow cytometry.

### Blocking Receptors

HL60 cells were cultured in IMDM supplemented with 20% fetal bovine serum. For differentiation into neutrophils, the cell medium was supplemented with 1.4% DMSO for 4 days, and the cells are thereafter referred to as neutrophils (29). The neutrophils, at 5X10^5^cells/100uL, were incubated with the following blocking antibodies: TREM-1, CD18, Galectin-3, Siglec-5, DPP4/CD26, CD40, IL-6 R, chitinase 3 like 1, L-selectin, CD93 and CXCL/IL-8 for 1 hr at 37°C and 5% CO_2_. They were then incubated with JP2 for 90min, stained with PI for 15 min on ice and analyzed with flow cytometry.

When stated, primary neutrophils from MIPP patients, instead of the differentiated HL60 cells, were isolated with Polymorphprep (Axis-shield).

### Statistical Analysis

Assays of neutrophil cytotoxicity, survival and ROS were performed in triplicate for eight subjects for each of the healthy and MIPP groups.

The data were analyzed with a statistical software package (SigmaStat; Jandel Scientific, San Rafael, CA, USA). One-way repeated measure analysis of variance (RM ANOVA) was applied to test the significance of the differences between the treated groups. If the results were significant, inter-group differences were tested for significance with Student’s *t* test and the Bonferroni correction for multiple testing.

## Results

### MIPP Neutrophils Are Resistant to JP2 Killing and Show Low Intracellular ROS Release

First, primary neutrophils from periodontally healthy, generalized periodontitis (stage III grade B) and MIPP subjects (n=3 for each group) were each incubated with *Porphyromonas gingivalis* (Pg), non-pathogenic (A. *actinomycetemcomitans*) or the MIPP pathogen A. *actinomycetemcomitans clone* (JP2). Following 90 min incubation, cells were collected for analysis of necrosis and bacterial clearance.

**Figure 1:**
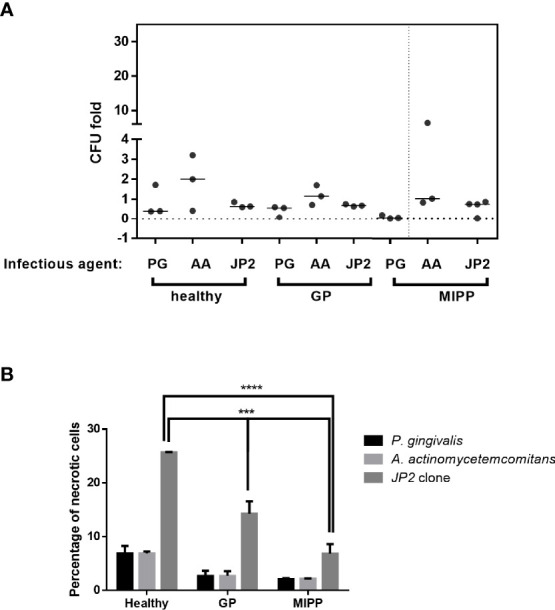
Necrosis of neutrophils exposed to various bacteria. Neutrophils isolated from three periodontally healthy cases, three generalized periodontitis (GP) cases and three molar incisor pattern periodontitis cases were incubated with *P. gingivalis* (PG), *A. actinomycetemcomitans* (AA) or the *A. actinomycetemcomitans* JP2 clone. **(A)** Colony forming units (CFU) as fold-CFU of the same bacterium without neutrophils. The horizontal bars represent the median for each group. **(B)** Percentage of necrotic neutrophils (propidium iodide-positive cells in flow cytometry analysis). The results are expressed as the mean ± standard error ***P<0.001; ****P<0.00001.

The results showed that JP2 and A. *actinomycetemcomitans* survival was similar in all the neutrophil groups (**Figure 1A**). Pg showed reduced survival in both periodontitis groups compared with that of the periodontally healthy group (**Figure 1A**). Greater resistance to neutrophil necrosis was observed in the MIPP group compared with that in the periodontally healthy and generalized periodontitis groups (**Figure 1B**, p<0.0001).

**Figure 2:**
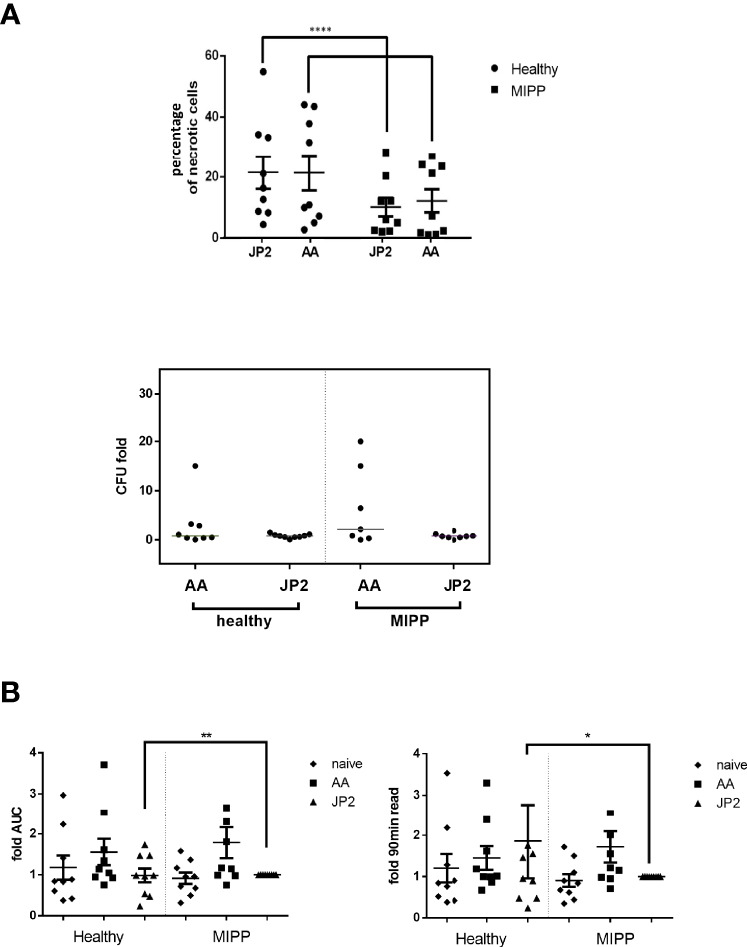
Necrosis and oxidative stress of neutrophils exposed to various bacteria. Neutrophils isolated from 10 periodontally healthy cases and 10 molar incisor pattern periodontitis cases were infected with *A. actinomycetemcomitans* (AA) or its JP2 clone (JP2). **(A)** Percentage of necrotic neutrophils (propidium iodide-positive cells in flow cytometry analysis). The results are expressed as the mean ± standard error. The horizontal bars represent the median; the T represents standard errors. Each shape represents a different patient. Colony forming units (CFU) as fold-CFU of the same bacterium without neutrophils. The horizontal bars represent the median for each group. **(B)** Fold-change of area under the curve (AUC) of total oxidative stress (as ROS production) and 90 min after bacterial inoculation. The data are presented as fold- JP2 MIPP as reference. The horizontal bars represent the median; T represents the standard error. The shapes represent the different patients. *P<0.05; **P<0.01; ****P<0.00001.

Based on the above results, the next experiment was conducted with MIPP and healthy control neutrophils (n=10 per group), and with A. *actinomycetemcomitans* and JP2 as infectious agents. The results were similar to those described in **Figure 1**, for both necrosis and bacterial survival (**Figure 2A**), suggesting a true trend in neutrophil behavior.

Next, we examined the reactivity of neutrophils to infection, by measuring intracellular reactive oxidative species (ROS). The only significant differences were between MIPP and healthy control neutrophils in response to JP2: MIPP neutrophils exhibited significantly lower levels of ROS production compared with that of the healthy neutrophils, both as cumulative ROS (as the area under the curve values, AUC) and as endpoint after 90 min bacterial infection (**Figure 2B**, p<0.001).

### The MIPP Neutrophil Cytokinome Is Substantially Different From That of Periodontally Healthy Neutrophils

**Figure 3:**
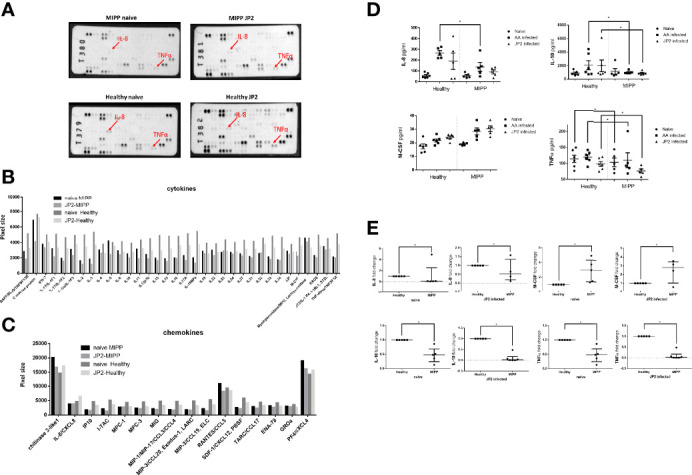
Secretome profiler quantification. The supernatants of neutrophils isolated from ten periodontally healthy cases and ten molar incisor pattern periodontitis (MIPP cases) that were incubated for 3hrs with *A. actinomycetencoomitans* (AA) or with the *A. actinomycetemcomitans* JP2 clone or were pooled. (equal volumes for each group) for the cytokine and chemokine secretion array. **(A)** Image of array membranes following analysis. The red arrows point to significant dots that show a change between groups. **(B)** Dot size quantification (in arbitrary values) of cytokines in the array for all four groups: MIPP with and without JP2 in comparison with healthy neutrophils with and without JP2. **(C)** Dot size quantification (in arbitrary values) of chemokines in the array for all four groups: MIPP with and without JP2 in comparison with healthy neutrophils with and without JP2. **(D)** ELISA quantification of IL-8, IL-10, M-CSF, TNF alpha for all case group supernatants (nonpooled samples). **(E)** Fold-change of real-time PCR quantification of IL-8, IL-10, M-CSF, TNF alpha for all case group supernatants (nonpooled samples). (*p < 0.05).

Secretome analysis was performed to corroborate the above findings and single out the unique cytokine pattern of MIPP versus periodontally healthy neutrophils. To that end, a profiler assay was used to map the neutrophil cytokine secretome (101 cytokines and chemokines, **Figure 3A** and **Supplementary Figure 1**).

MIPP naïve and JP2 infected neutrophils expressed reduced inflammatory levels of most of the tested cytokines compared with those of periodontally healthy neutrophils (**Figure 3B**). Chemokine expression levels also showed reduced expression in MIPP neutrophils compared with those in periodontally healthy neutrophils (**Figure 3C**).

As the profiler assay is a semi-quantitative assay, we further quantified selected cytokines (IL-8, IL-10, M-CSF, and TNFα) using ELISA and RT-PCR. The ELISA results (in pg/ml) showed significantly reduced levels of the above cytokines in MIPP neutrophils (**Figure 3D**), which were corroborated by qRT-PCR (**Figure 3E**).

### JP2 Adheres Extracellularly to the Neutrophil Cell Membrane to Induce Necrosis

Next, we examined the nature of the interaction between JP2 and neutrophils.

**Figure 4:**
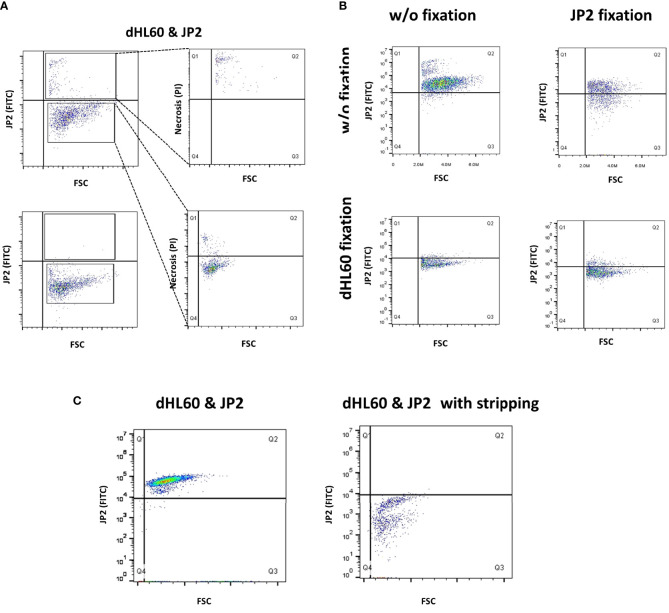
Interaction between JP2 and HL-60 cells. Differentiated HL-60 cells were infected with the JP2 clone of *A. actinomycetencoomitans* (JP2) for analysis of the interaction between the cells and the bacteria. Gating strategy is displayed in **Supplementary Figure 3**. **(A)** The site of JP2 adherence to neutrophils was examined by staining JP2 with fluorescein isothiocyanate (FITC), followed by quenching of the extracellular FITC staining with trypan blue, and analysis with flow cytometry. the top plot show analysis without quenching; the bottom plot show analysis with quenching. **(B)** Examination of JP2 adherence to neutrophils by denaturation of protein (paraformaldehyde fixation) and the use of fluorescein isothiocyanate (FITC)- tagged JP2. **(C)** Examination of JP2 adherence to neutrophils following incubation with stripping protein and the use of fluorescein isothiocyanate (FITC)-tagged JP2.

Using the human cell line HL60 and JP2 for this purpose, we examined whether the bacterium adheres to the neutrophils and if so, whether the interaction occurs extracellularly or intracellularly. dHL60 cells were incubated with fluorescein isothiocyanate- (FITC) stained JP2 (29). Trypan blue was used to quench extracellular FITC staining. Gating strategy is presented in **Supplementary Figure 3**. The results showed that the dHL60 were all positive for FITC staining, and the FITC-stained cells were also positive for necrosis (PI, **Figure 4A**). Trypan blue abrogated the FITC signal on the dHL60, suggesting that the bacteria adhere to the cells extracellularly.

We next examined whether the interaction between JP2 and the neutrophils is protein-mediated. We denaturized the surface proteins (by fixation with paraformaldehyde) on the neutrophils and/or JP2 before the infection. The results showed that denaturation of the proteins on neutrophils abrogated JP2 attachment, whereas the same process on JP2 did not (**Figure 4B**). Furthermore, stripping off the inter-protein association (with a stripping buffer) detached the JP2 from the neutrophils (**Figure 4C**).

### Neutrophil Receptor Immunoprecipitation Displays an Array of Proteins That Adhere to JP2

**Figure 5:**
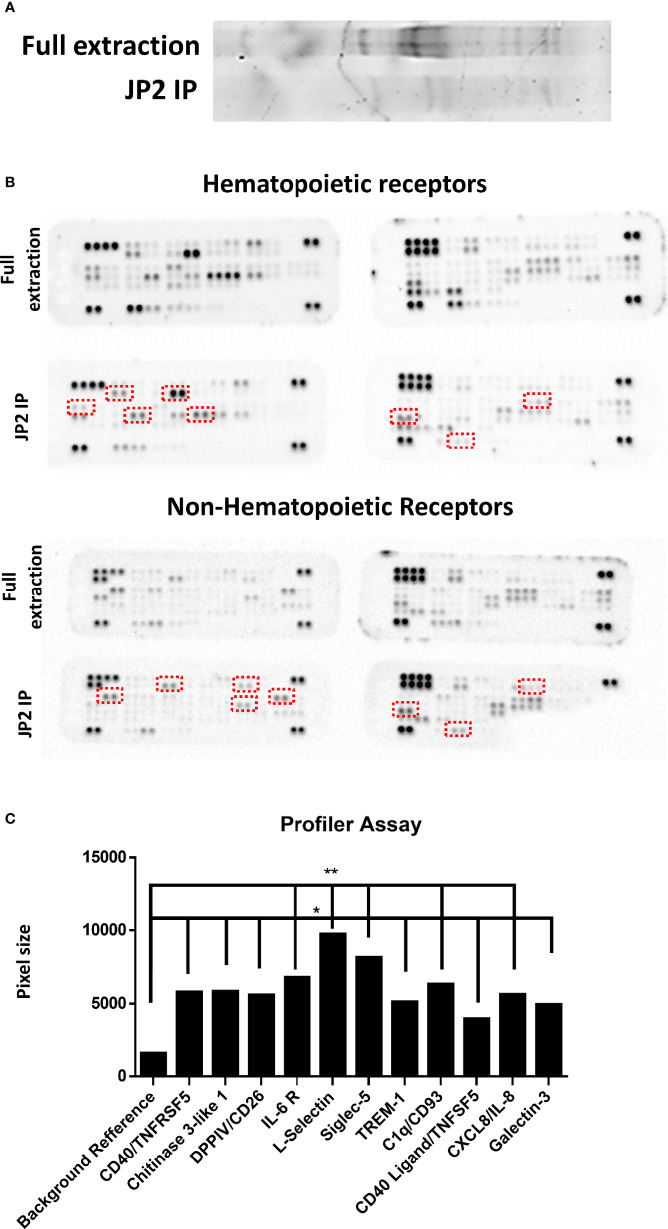
Profiler Quantification. Wide-range receptors analysis. Proteins adhering to JP2 were isolated with an immunoprecipitation (IP) assay and analyzed with receptor array assays. **(A)** Gel separation of proteins of the full extract from healthy neutrophils and after JP2 IP. **(B)** Image of array membranes (hematopoietic and non-hematopoietic receptors) following analysis. The red squares mark significant dots that show a change between groups. **(C)** Dot size quantification (in arbitrary values) of receptors showing enrichment compared with the total protein level as reference point. (*p< 0.05, **<0.001).

Next, a wide receptor profile was made in order to single out potential receptors that interact with JP2. A lysate of neutrophils (dHL60) was incubated with JP2, and then the JP2s (with the attached proteins from the cell lysate) were collected, the inter-protein association was stripped, and the JP2 was removed by filtration. The extracted proteins showed specific targets compared with the entire neutrophil lysate (**Figure 5A**). Also, the specific protein levels were higher than the whole extraction samples (**Figure 5B**) (for those most enriched by the JP2 immunoprecipitation see **Table 1** and **Figure 5C**).

**Table 1:** Enriched receptors.

Receptor name	Enrichment Fold (JP2 IP vs full extraction)	Known function	Reference
**TREM-1**	2.25 (44%)	The triggering receptor expressed on myeloid cells 1 (TREM-1) is an immunoglobulin superfamily transmembrane receptor found mostly on neutrophils, monocytes, and macrophages. TREM-1 triggers and amplifies inflammatory immune responses by interacting with Toll-like receptor signaling. Recently TREM-1 has been suggested as a candidate marker for new treatment strategies against periodontal diseases.	(30, 31)
**Integrin β2/CD18**	1.66 (59.9%)	Beta2-integrins are complex leukocyte-specific adhesion molecules that are required for leukocyte (e.g., neutrophil, lymphocyte) trafficking as well as other immunological activities such as neutrophil phagocytosis and formation of reactive oxygen species (ROS) and T cell activation.	(32)
**Galectin-3**	1.49 (66%)	Galectin-3 is a lectin that binds to -galactosides and plays a vital function in a variety of biological processes. Galectin-3 can influence the composition of the microbial community in the oral cavity by directly attaching to microorganisms and modulating their clearance. Galectin-3 also affects immunological homeostasis by modulating the function of numerous immune cells in the gingiva and gingival sulcus.	(33)
**Siglec-5**	1.81 (54.1%)	SIGLECs (sialic acid-binding immunoglobulin-like lectins) are immunoglobulins that facilitate protein-carbohydrate interactions. Siglec 5 protein is found mostly in neutrophils and performs a regulatory role in myeloid cell activation in order to prevent improper reactivity against self-tissues.	(34, 35)
**DPPIV/CD26**	1.96 (50%)	CD26 is a highly glycosylated type II membrane sialoglycoprotein composed of two identical subunits of approximately 110 kDa and is a cell surface ectoenzyme, dipeptidylpeptidase IV (DPPIV). CD26/DPPIV has a unique specificity: if proline is in the penultimate position, it cleaves dipeptides from the N terminus of a polypeptide. If alanine or hydroxyproline is present in the next (P1) position, peptides are also cleaved.	(36, 37)
**CD40/TNFRSF5**	2.43 (41.1%)	CD40 is a transmembrane protein with a molecular weight of 50 kDa that regulates lymphocyte proliferation and differentiation. Hematopoietic cells, such as lymphocytes, follicular dendritic cells, and monocytes, are the main producers of this new activation antigen. CD40 was recently discovered in human fibroblasts from a variety of tissues, but its function was unknown. The CD40 ligand, which is found on activated cells, mast cells, eosinophils, basophils, and lineage cells, is a natural trigger for cellular responses mediated by CD40.	(38)
**IL-6 R**	2.58 (38.7%)	The cytokine interleukin-6 (IL-6) is involved in the control of the host response to bacterial infection. Importantly, differentiated THP-1 cells treated with IL-6 generate sIL-6R, which is required for IL-6 signaling in HGFs.	(39)
**Chitinase 3-like-1**	1.17 (85.2%)	(CHI3L1) is also known as Cartilage glycoprotein 39 (CGP-39), YKL-40, which belongs to the glycosyl hydrolase 18 family. The protein lacks chitinase activity and is secreted by activated macrophages, chondrocytes, neutrophils and synovial cells. Chitinase-3-like-1 (Chi3l1) is known to play a significant role in the pathogenesis of Type 2 inflammation and cancer	(40)
**L-selectin**	2.38 (41.8%)	L-selectin is a selectin family member that is expressed on all leukocytes, including peripheral lymphocytes, monocytes, and neutrophils.	(41)
**C1q/CD93**	2.33 (42%)	CD93 is a member of a newly discovered transmembrane glycoprotein family that also contains endosialin and thrombomodulin. The ectodomain architecture of these cell surface proteins is similar, with a C-type lectin-like domain, a series of EGF-like repeats, and a heavily glycosylated mucin-like domain.	(42)
**CXCL8/IL-8**	2.67 (37.3%)	Interleukin-8 (IL-8) is a chemokine that has been linked to periodontal disease. Endothelial cells, gingival fibroblasts, neutrophils, monocytes, and phagocytes in the gingival crevice generate IL-8, a powerful chemoattractant cytokine and neutrophil activator in inflammatory regions.	(43)

**Figure 6:**
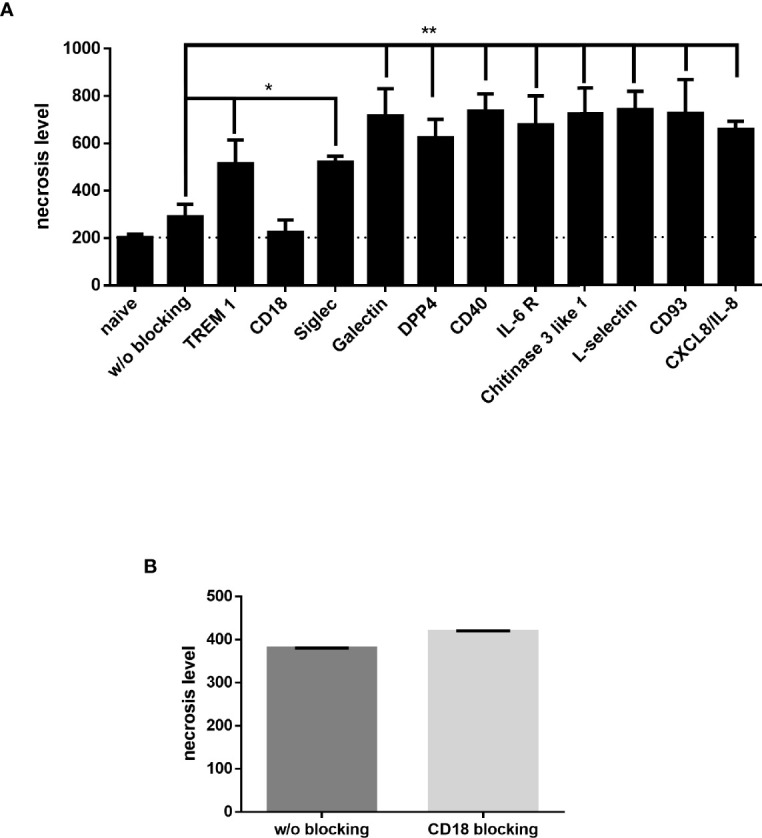
Neutrophil blocking receptors. Neutrophils blocked with specific antibodies were incubated with JP2 and cell necrosis (propidium iodide-positive cells in flow cytometry) was examined. **(A)** Necrosis level of differentiated HL60 blocked with specific antibodies (*p< 0.05, **<0.001). **(B)** Necrosis level of MIPP neutrophils blocked with CD18.

The enriched receptors (TREM-1, CD18, Galectin-3, Siglec-5, DPP4/CD26, CD40, IL-6 R, chitinase 3-like 1, L-selectin, CD93 and CXCL/IL-8) were then functionally tested by blocking each one before JP2-dHL60 interaction and cell necrosis was examined. Of the tested receptors, only blocking of CD18 rescued the neutrophils from necrosis upon infection with JP2 (**Figure 6A**, P<0.05).

Finally, in order to validate that CD18 indeed has a role in the necrosis resistance of the MIPP neutrophils, CD18 on primary neutrophils from MIPP cases was blocked and then challenged with JP2 and neutrophil necrosis was examined. Indeed, in comparison with the necrosis rescue effect in healthy neutrophils by CD18 blocking (**Figure 6A**), the same procedure did not affect MIPP neutrophil necrosis, showing that the CD18 in these cells is functionally different from that in periodontally healthy neutrophils (**Figure 6B**).

## Discussion

Neutrophils are one of the first lines of defense in the immune response to periodontal infection. Evidence shows that in MIPP these cells have altered functions, concomitant with a persistent infection by the JP2 clone of *A*. *actinomycetemcomitans* (29). The present study portrays an interesting characterization of MIPP neutrophils: while unable to clear the JP2 pathogen, these cells do not die (*via* necrosis) or respond to the pathogen, compared with periodontally healthy neutrophils.

Current knowledge demonstrates conflicting results regarding the nature of the communication between JP2 and MIPP neutrophils. Some maintain that the JP2 clone increases neutrophil cytotoxicity (44), whereas others show a reduced neutrophil response to JP2, leading to periodontal tissue damage. However, the unique interaction between A. *actinomycetemcomitans* and MIPP neutrophils has been established (45, 46), revealing a fundamental difference in the response to periodontal bacterial infection between healthy and MIPP neutrophils (47, 48), in immune system recruitment *via* cytokine/chemokine levels, or ROS production levels and phagocytosis ability (48, 49). In our study this was manifest by MIPP neutrophil resistance to necrosis and lowered ROS and cytokine production, despite the persistent presence of viable JP2. Also, the refractory nature of MIPP neutrophils responses to JP2 was manifested by the low expression of inflammatory cytokines, compared with that in periodontally healthy neutrophils. While the current study found that JP2 is not phagocytized by periodontally healthy neutrophils, previous evidence show that neutrophils do internalize opsonized-JP2 but at greater multiplicity of infection the one used in the current study (50); the current study did not compared phagocytosis in the different periodontal cases which may also be affected by CD18 and result in differences in phagocytosis functionality. Overall, our results suggest a specific lack of responsiveness of MIPP neutrophils to JP2, which may be attributable to poor pathogen recognition. Other studies have also demonstrated NETosis as a key player in neutrophils function (51), albeit this trait was not examined in the current study.

The extracellular region of human CD18 is important for the human specificity of LtxA-induced cell lysis in humans and for conferring susceptibility to LtxA-induced cell lysis (17, 52). Our results also show that although CD18 is a key mediator of the healthy neutrophil response to JP2, blocking this receptor has no impact in MIPP neutrophils, suggesting a unique interaction between the pathogen and MIPP neutrophil CD18. Moreover, whilst numerous proteins bind to JP2 (such as TREM-1, CXCL-8, L-selectin), only CD18 appears to play a significant role in JP2-mediated necrosis of human neutrophils. The study also shows that JP2-induced neutrophil necrosis is mostly mediated by direct contact between the bacterium and the neutrophils, suggesting that the bacterial` cell wall-bound Ltx is involved to a greater extent than its secreted form.

Overall, our study shows that MIPP neutrophils react differently to exposure to JP2 in comparison with healthy patient neutrophils, leading to reduced necrosis, lower levels of ROS production and reduced levels of inflammatory cytokines. This may be one of the reasons for host susceptibility of MIPP patients to JP2 and may play a fundamental role in MIPP pathogenesis. The finding of the current study paves the path for understanding the mechanism of neutrophil activation in MIPP, which in turn, can be further developed to early diagnosis and more specific therapeutic modalities.

## Data Availability Statement

The raw data supporting the conclusions of this article will be made available by the authors, without undue reservation.

## Ethics Statement

The studies involving human participants were reviewed and approved by Hadassah Medical Center IRB. The patients/participants provided their written informed consent to participate in this study.

## Author Contributions

KH, WA, SD, and DP have made substantial contributions to conception and design, or acquisition of data, or analysis and interpretation of data; IC, LS, and DP have been involved in drafting the manuscript or revising it critically for important intellectual content; and KH, IC, LS, WA, SD, and DP have given final approval of the version to be published. Each author participated sufficiently in the work to take public responsibility for appropriate portions of the content; and DP agreed to be accountable for all aspects of the work in ensuring that questions related to the accuracy or integrity of any part of the work are appropriately investigated and resolved.

## Conflict of Interest

The authors declare that the research was conducted in the absence of any commercial or financial relationships that could be construed as a potential conflict of interest.

## Publisher’s Note

All claims expressed in this article are solely those of the authors and do not necessarily represent those of their affiliated organizations, or those of the publisher, the editors and the reviewers. Any product that may be evaluated in this article, or claim that may be made by its manufacturer, is not guaranteed or endorsed by the publisher.
