# Supplementary material for: CD18 Mediates Neutrophil Imperviousness to the Aggregatibacter actinomycetemcomitans JP2 Clone in Molar-Incisor Pattern Periodontitis
Source: Front Immunol. 2022 May 18;13:847372. doi: 10.3389/fimmu.2022.847372 (PMC9159298; doi:10.3389/fimmu.2022.847372)
Supplement: Supplementary file 1 [file Presentation_1.pptx]

## Slide 1
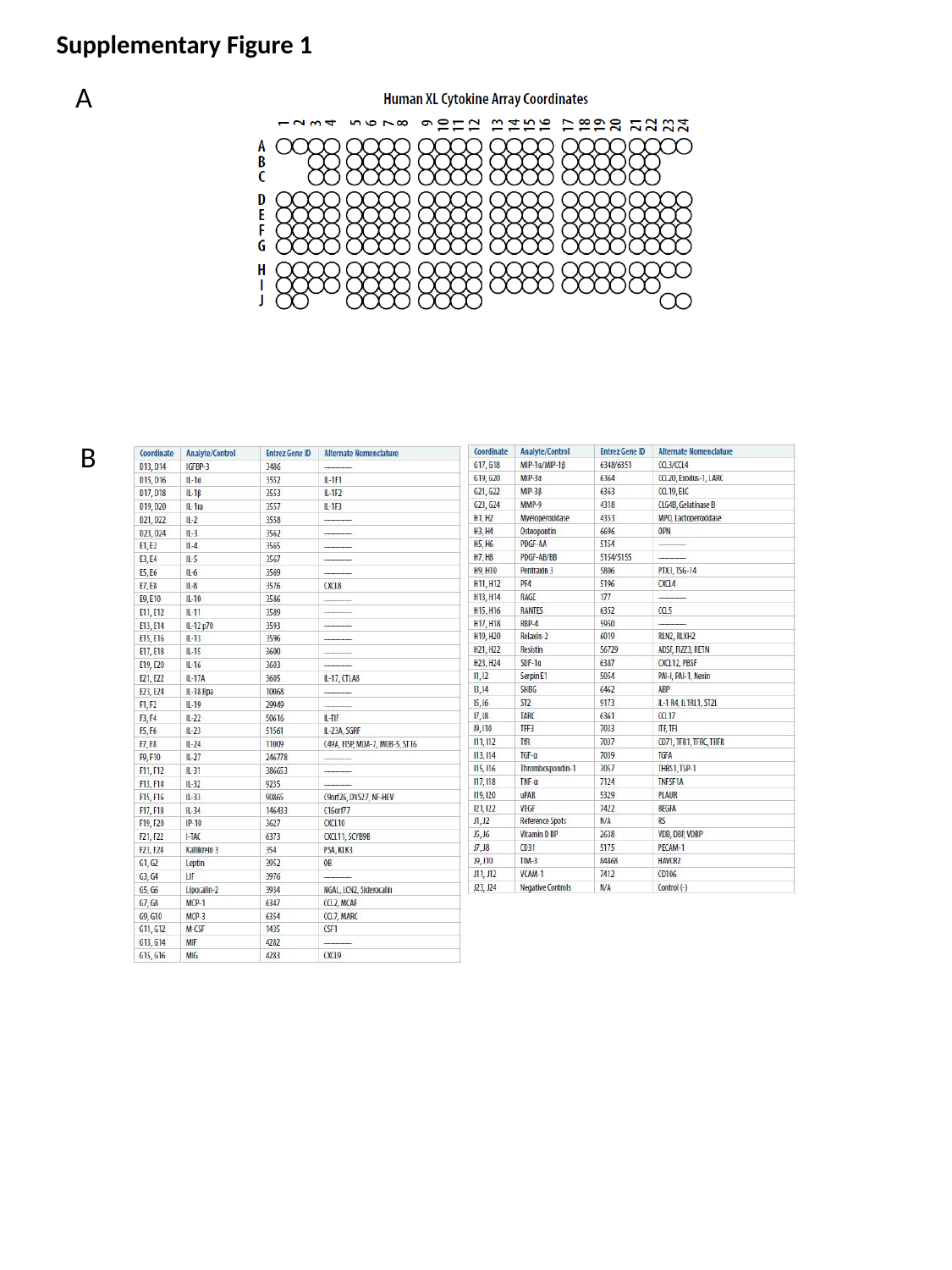

Supplementary Figure 1
A
B

## Slide 2
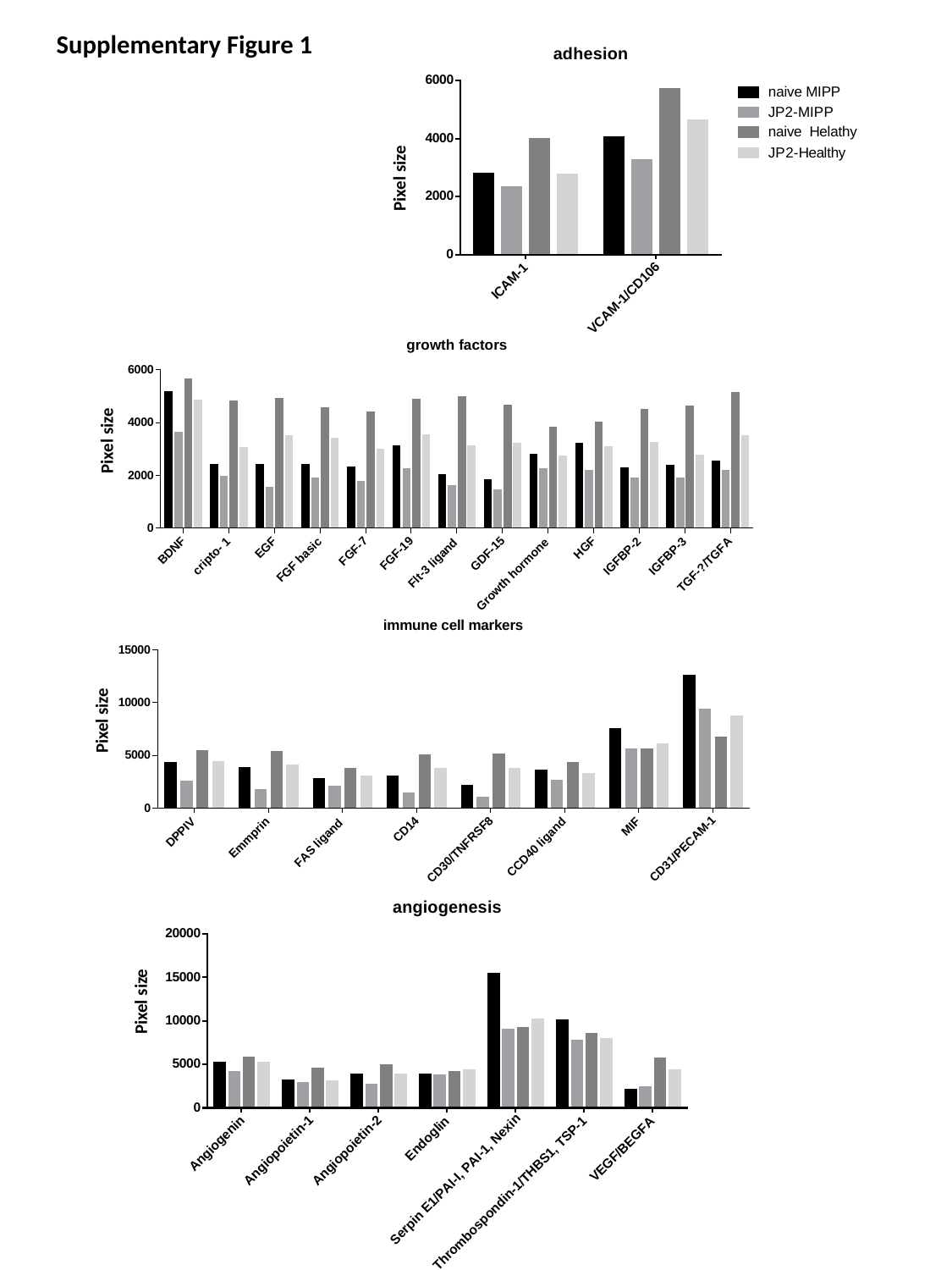

Supplementary Figure 1
Pixel size
Pixel size
Pixel size
Pixel size

## Slide 3
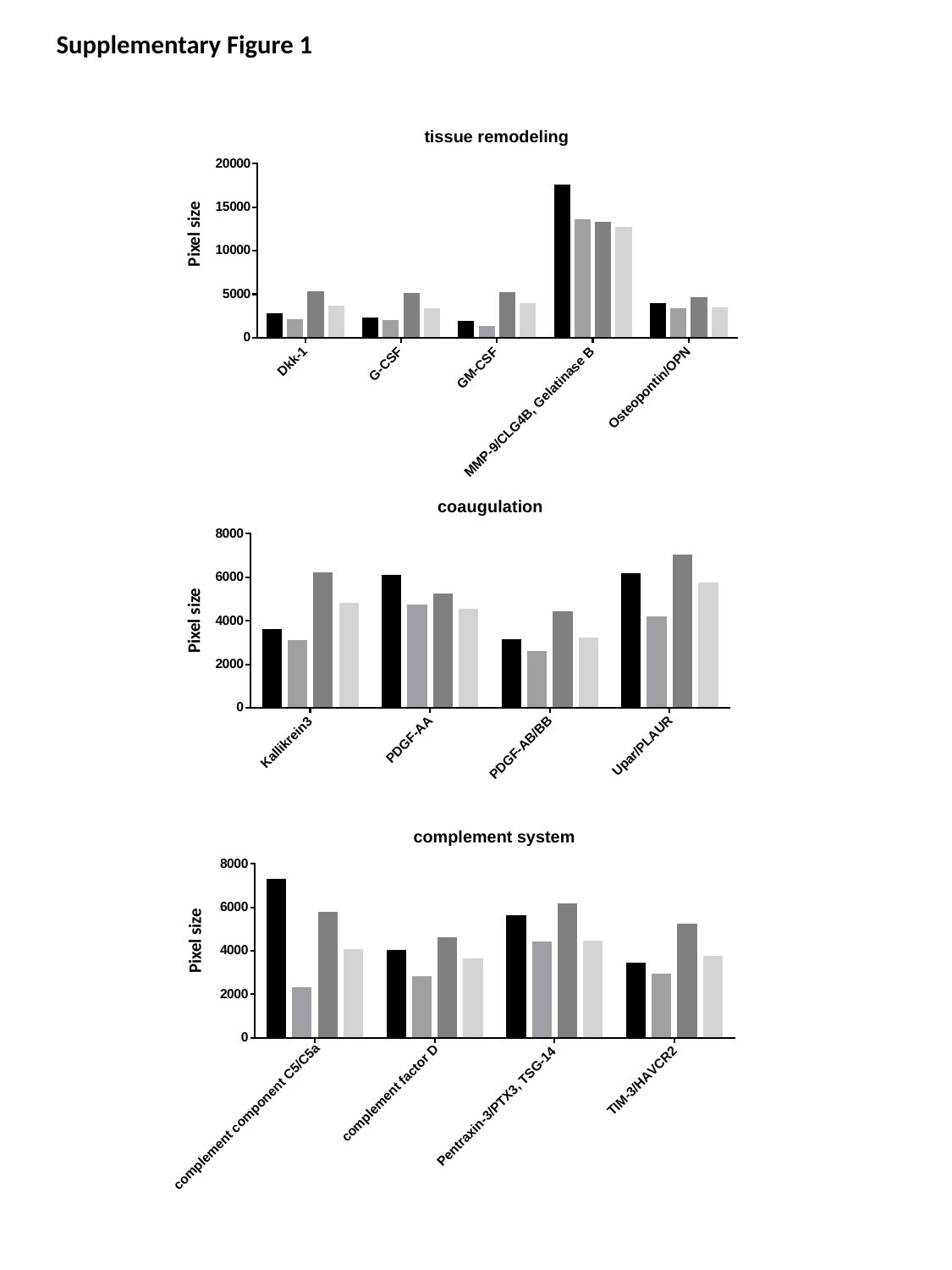

Supplementary Figure 1
Pixel size
Pixel size
Pixel size

## Slide 4
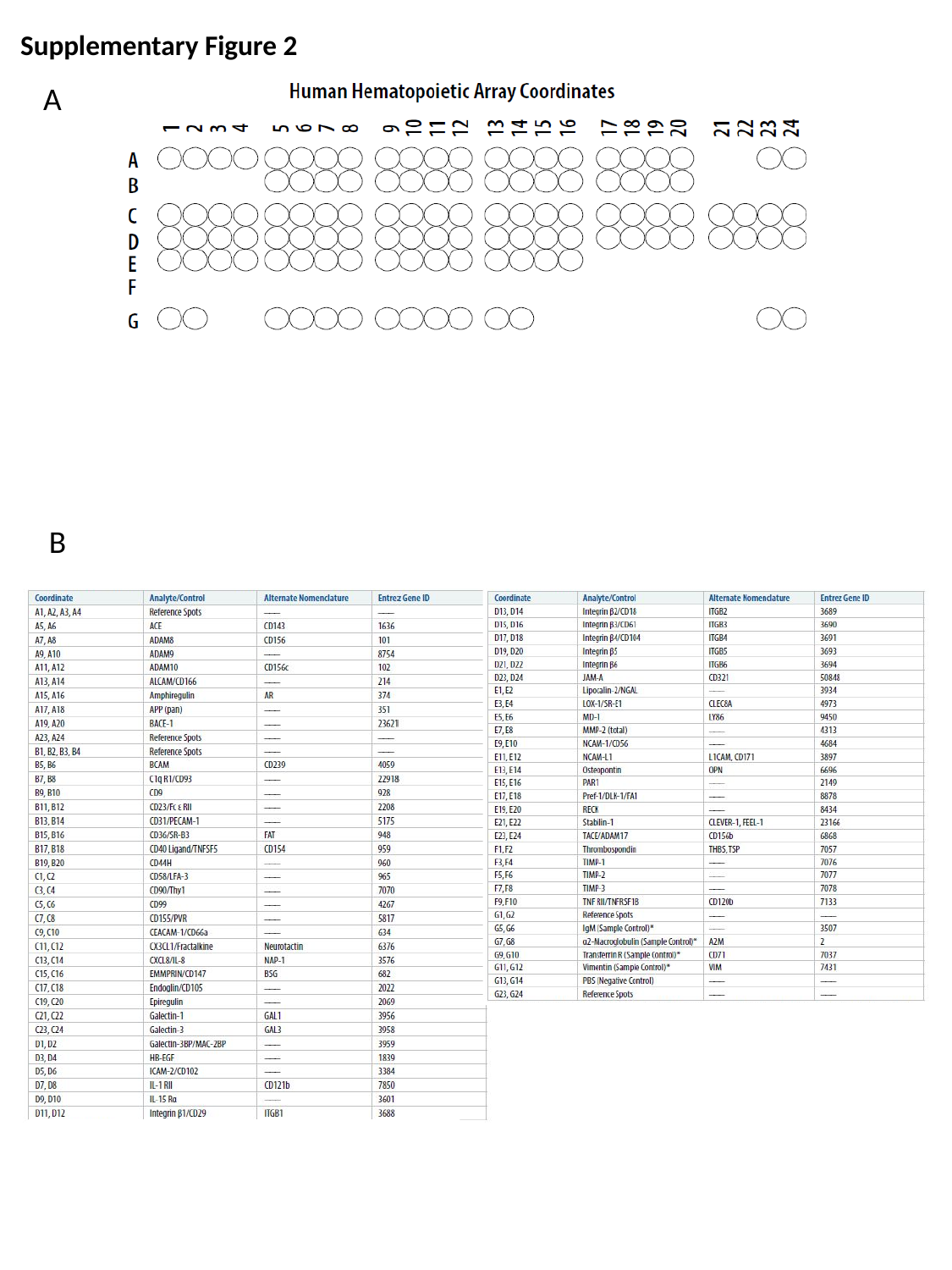

Supplementary Figure 2
A
B

## Slide 5
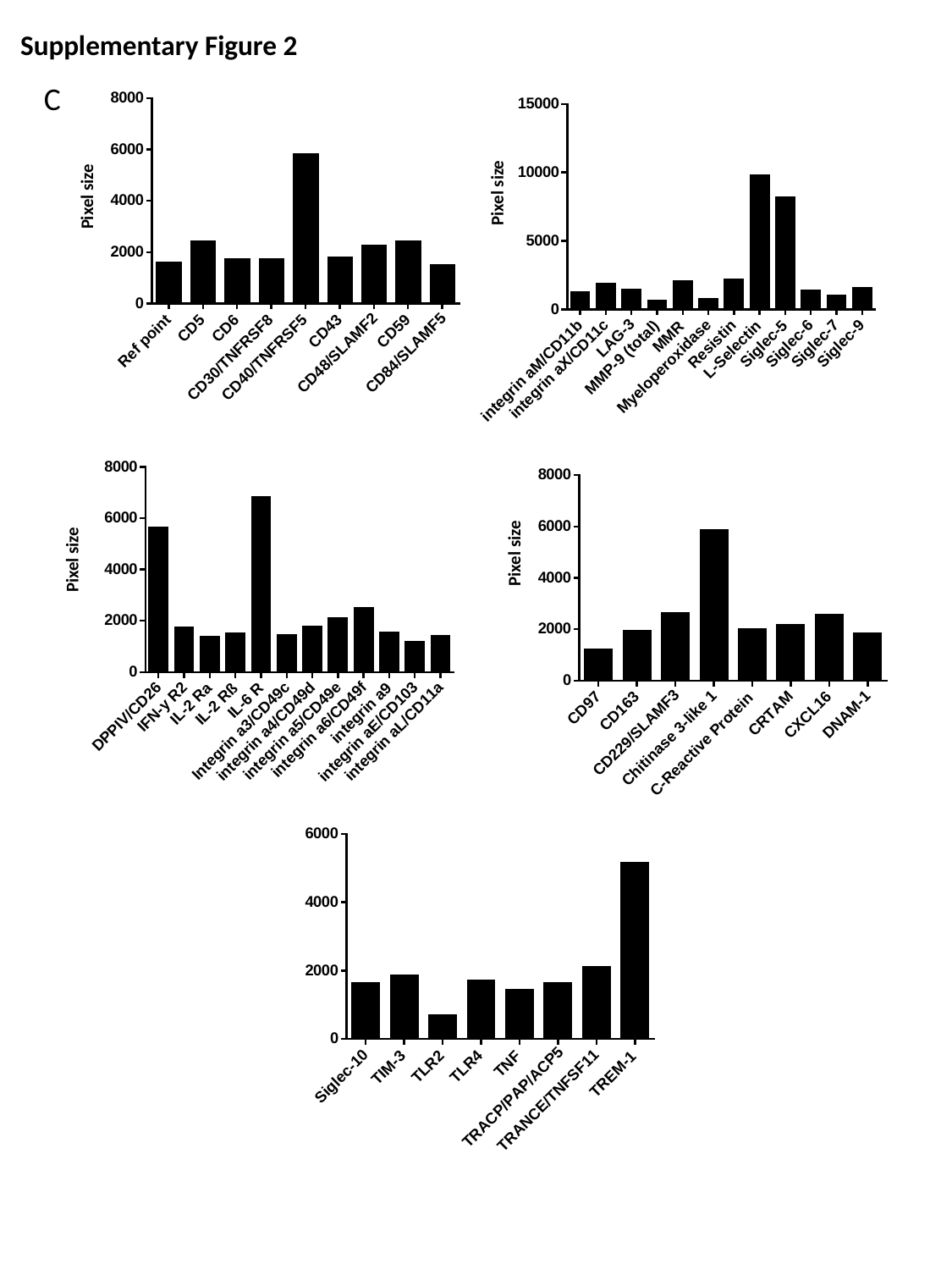

Supplementary Figure 2
C
Pixel size
Pixel size
Pixel size
Pixel size

## Slide 6
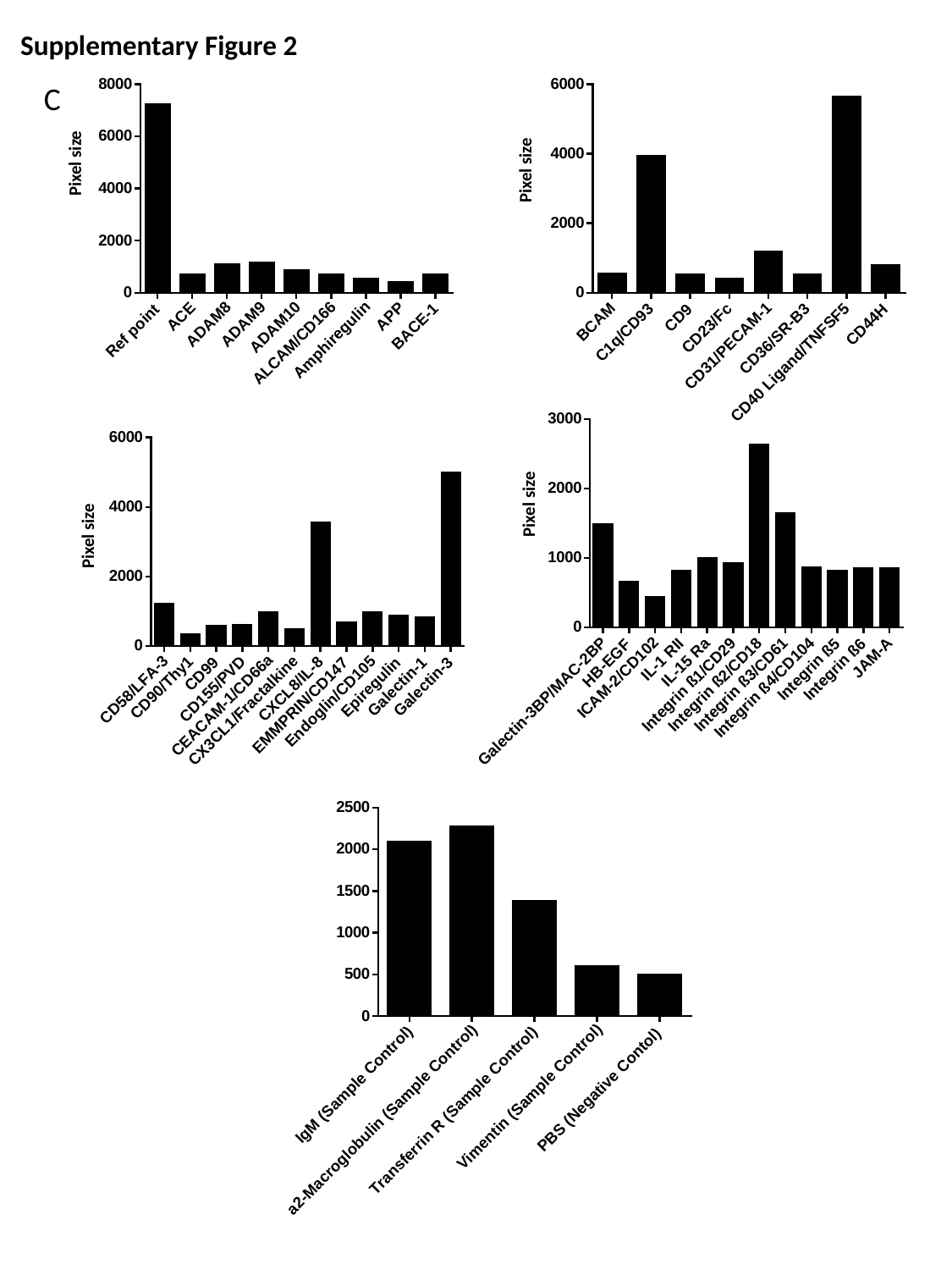

Supplementary Figure 2
C
Pixel size
Pixel size
Pixel size
Pixel size

## Slide 7
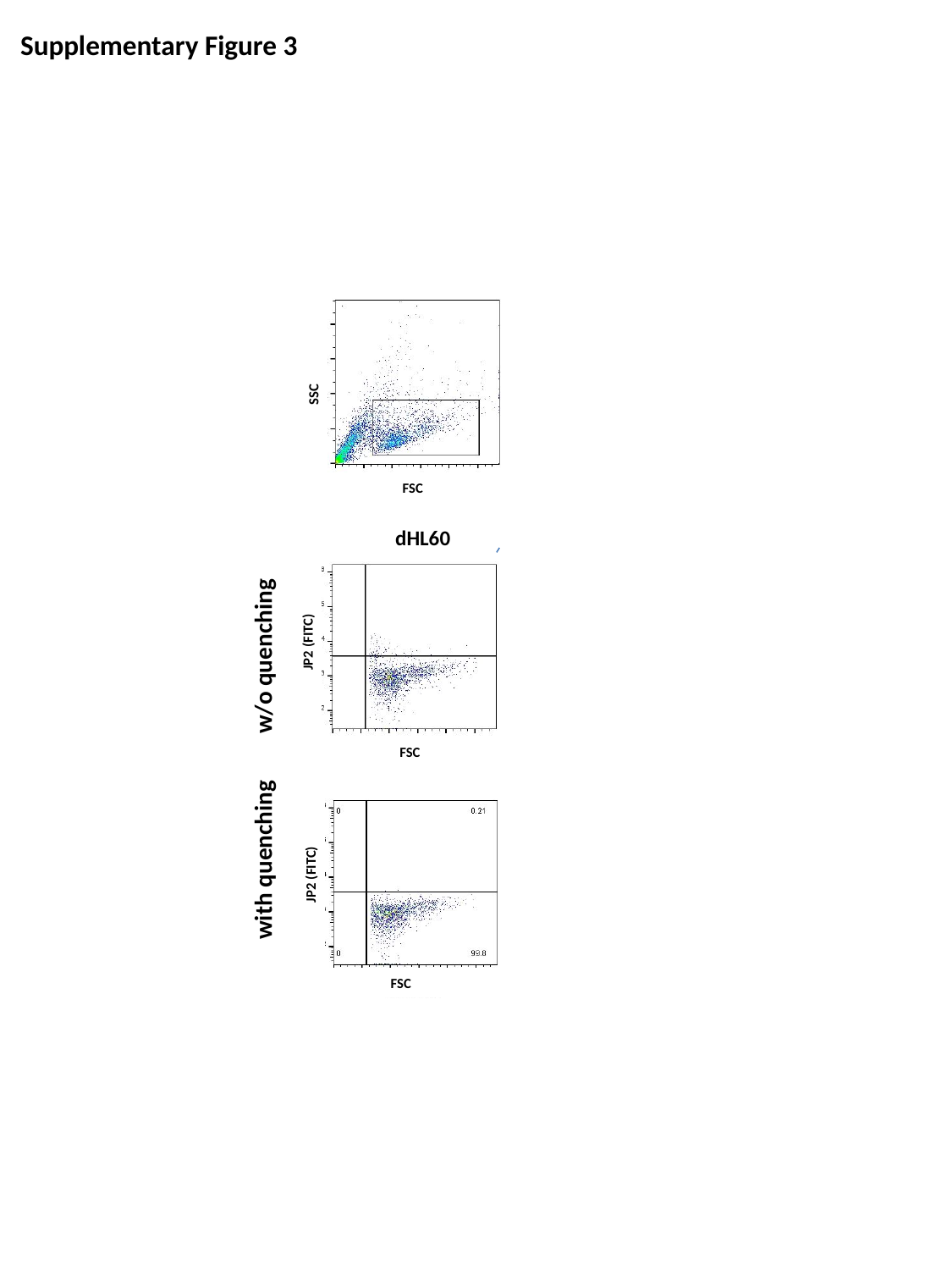

Supplementary Figure 3
SSC
FSC
dHL60
JP2 (FITC)
w/o quenching
FSC
with quenching
JP2 (FITC)
FSC
